# Aging impairs dendrite morphogenesis of newborn neurons and is rescued by 7, 8‐dihydroxyflavone

**DOI:** 10.1111/acel.12553

**Published:** 2017-03-03

**Authors:** Xiaoting Wang, Jennifer Lynn Romine, Xiang Gao, Jinhui Chen

**Affiliations:** ^1^ Spinal Cord and Brain Injury Research GroupStark Neuroscience Research InstituteIndianapolisIN46202USA; ^2^ Department of Neurological SurgeryIndiana University School of MedicineIndianapolisIN46202USA

**Keywords:** aging, brain‐derived neurotrophic factor signaling, dendritic morphology

## Abstract

All aging individuals will develop some degree of decline in cognitive capacity as time progresses. The molecular and cellular mechanisms leading to age‐related cognitive decline are still not fully understood. Through our previous research, we discovered that active neural progenitor cells selectively become more quiescent in response to aging, thus leading to the decline of neurogenesis in the aged hippocampus. Here, we further find that aging impaired dendrite development of newborn neurons. Currently, no effective approach is available to increase neurogenesis or promote dendrite development of newborn neurons in the aging brain. We found that systemically administration of 7, 8‐dihydroxyflavone (DHF), a small molecule imitating brain‐derived neurotrophic factor (BDNF), significantly enhanced dendrite length in the newborn neurons, while it did not promote survival of immature neurons, in the hippocampus of 12‐month‐old mice. DHF‐promoted dendrite development of newborn neurons in the hippocampus may enhance their function in the aging animal leading to a possible improvement in cognition.

## Introduction

Aging is a process in which overall body functions fall over time. As we grow older, we can experience memory loss and cognitive decline, including processing speed decrease, working memory deficits and episodic memory encoding dysfunctions, which can interfere with our daily routines (Hedden & Gabrieli, 2004). According to statistics from U.S. Census Bureau, by 2050, the older population, which is over 65‐year‐old, is estimated to reach 83.7 million, accounting for around 20% of the total population (Ortman *et al*., 2014). A treatment approach is urgently needed to target aging‐related cognition disorders. However, the molecular and cellular mechanisms of age‐dependent cognitive decline remain elusive, impeding the development of an effective clinical treatment.

Hippocampal neurogenesis and synaptic plasticity, which are involved in cognitive functions, are both greatly affected by the aging process (Rao *et al*., 2006). Neurogenesis comprises several critical steps, including neural stem/progenitor cell (NSC) proliferation, immature neuron survival, dendrite development, maturation, and functional integration (Ming & Song, 2005). Our previous study proved that aging specifically reduces proliferation of active neural progenitors (Romine *et al*., 2015). Additionally, the consistently low survival rate of newborn neurons further impedes the production of newborn neurons that can successfully integrate into existing neuronal circuitry in the aging animals (Bondolfi *et al*., 2004; Rao *et al*., 2005).

After new neurons were generated in the hippocampus, morphological development and synaptic integration are required for appropriate function. The morphological maturation serves as a foundation for granule neurons to properly relay signal inputs received by dendritic spines to the CA3 region by means of axons. In the adult hippocampal dentate gyrus, newborn neurons generally grow proper axonal and dendritic morphology and spine structure in around 3–4 weeks of birth, while further structural modification lasts for months (Zhao *et al*., 2006). Here, we found that aging, besides compromising neurogenesis, significantly impairs dendrite development through diminishing dendritic branches and total dendrite length. As dendrites provide a large surface for synapse formation, reduction in dendritic branches and total dendrite length ultimately damages synaptogenesis. So far, no effective approach is available to prevent or rescue neurogenesis and dendrite development from impairments during aging.

Both neurogenesis and morphological maturation of adult‐born neurons are known to be regulated by brain‐derived neurotrophic factor (BDNF), which functions through binding and activation of its specific receptor, tropomyosin‐related kinase receptor B (TrkB) (Tolwani *et al*., 2002; Binder & Scharfman, 2004). The downstream effects of BDNF promote neuronal growth, survival, migration and synapse modulation (Alderson *et al*., 1990; Horch & Katz, 2002; Gorski *et al*., 2003; Cohen‐Cory *et al*., 2010). In particular, BDNF plays an important role in regulating dendrite development and later functional and structural synaptic plasticity in the hippocampus (Tolwani *et al*., 2002; Vigers *et al*., 2012; Wang *et al*., 2015) and, thus, is functionally involved in memory formation and consolidation (Vigers *et al*., 2012). However, the BDNF‐TrkB system is particularly sensitive to the effects of aging, which may help explain the age‐related decline in hippocampal‐dependent memory (Calabrese *et al*., 2013; Budni *et al*., 2015) and provide a therapeutic target for improving memory function in the aging population. Although BDNF is a vital neurotrophic factor, its limited delivery, short half‐life and inability to cross the blood–brain barrier hinder its effectiveness as a therapeutic agent. Alternatively, 7,8‐dihydroxyflavone (DHF), a small molecule that can mimic BDNF to activate TrkB receptors (Jang *et al*., 2010), is advantageous, because it can cross the blood–brain barrier, is unlikely to cause an immune response, and can be noninvasively administered via intramuscular (i.m.), intraperitoneal (i.p.), or intravenous (i.v.) injections.

Our previous studies proved that DHF, as a highly potent BDNF receptor agonist, can promote neurogenesis and dendrite development in the hippocampus of adult mice in the case of traumatic brain injury (Zhao *et al*., 2015), indicating the beneficial effects of DHF. In this study, we sought to determine whether DHF benefits aged mice by promoting newborn neuron survival and their dendrite development in the hippocampus.

## Results

### Aging dramatically reduces the number of newborn immature neuron and impairs dendritic morphology in the hippocampus

**Figure 1:**
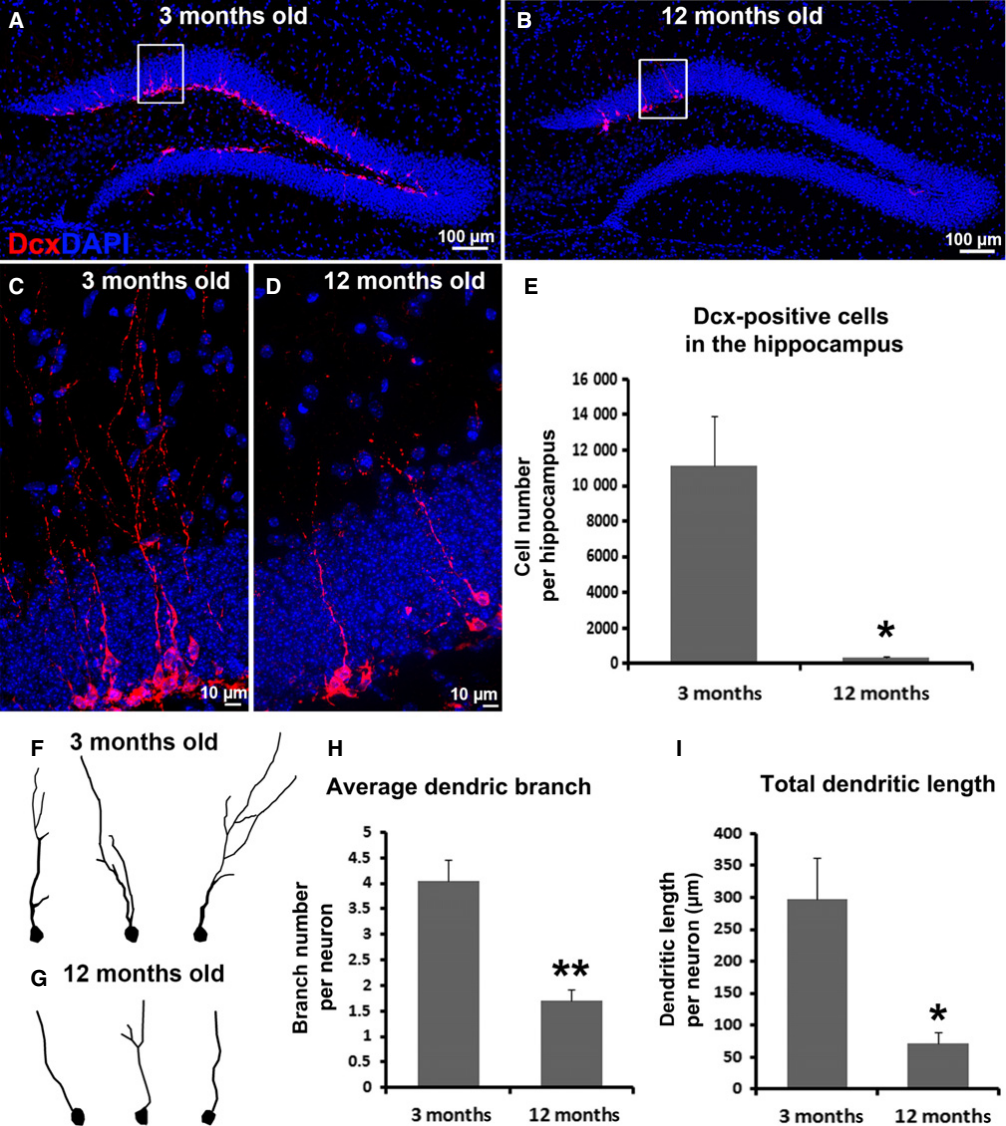
Aging decreases immature neuron number and impairs dendritic morphology development in newborn immature neurons in the hippocampus in aged mice. (A, B) Immunostaining with antibody against doublecortin (Dcx, red) shows immature neurons in the hippocampal dentate gyrus (HDG) of 3‐month‐old (A) and 12‐month‐old mice (B). 4′,6‐diamidino‐2‐phenylindole (DAPI, blue) staining shows the structure of HDG. (C‐D) Image of higher magnification shows structure of Dcx‐positive immature neurons in A and B (indicated by white boxes). (E) Quantification of Dcx‐positive newborn immature neuron number in the HDG of 3‐month‐old (*n* = 3) and 12‐month‐old (*n* = 4) mice. (F, G) Reconstruction of dendrites from Dcx‐positive newborn immature neurons in the HDG of 3‐month‐old (F) and 12‐month‐old (G) mice. (H, I) Quantification of average dendritic branch number (H), and total dendritic length (I) of Dcx‐positive immature neurons in the HDG of 3‐month‐old and 12‐month‐old mice. (*P < 0.05, **P < 0.01).

Cognitive decline is a hallmark of the aging process, while the mechanism remains elusive (Bishop *et al*., 2010). Among the potential mechanisms, decreased neuroplasticity in the hippocampus represented by neurogenesis reduction and synapses loss is an important contributor (Burke & Barnes, 2006). Neurogenesis, including NSC proliferation, immature neuron survival, migration, maturation, dendritic development, and functional integration (Ming & Song, 2005), is affected by aging at several levels. Our previous study demonstrated that aging impairs neurogenesis largely by compromising NSC, especially active neural progenitors, proliferation in the hippocampus in mice (Romine *et al*., 2015). Here, we further assessed the number of newborn immature neurons and their dendrite development in the 3‐month‐old (*n* = 3) and 12‐month‐old (*n* = 4) mice. Series of every sixth brain sections were processed for immunostaining with antibody against doublecortin (Dcx), a newborn immature neuron marker (Gleeson *et al*., 1999; Fig. S1, Supporting information). In 3‐month‐old mice, Dcx‐positive newborn immature neurons distributed mainly in the inner one‐third of the granule cell layer (GCL) of the hippocampal dentate gyrus (HDG) (Fig. 1A). In comparison with 3‐month‐old adult mice, we observed an obvious reduction in newborn immature neuron number with very few Dcx‐positive cells sporadically located in the inner one‐third of GCL in the HDG of 12‐month‐old mice (Fig. 1B). At higher magnification, we can clearly see each individual Dcx‐positive cell located at the inner granule cell layer (Fig. 1C,D). There were 11 098 ± 2841 newborn immature neurons per HDG in the 3‐month‐old mice, but this number reduced to 341 ± 54 in the 12‐month‐old mice, indicating a dramatic reduction in the number of newborn immature neurons in the aged animal (*P* = 0.022, Fig. 1E).

At higher magnification, dendrites of immature neurons were also clearly revealed growing toward molecular layer (Fig. 1C,D). Dcx‐positive cells in the 12‐month‐old mice exhibited decreased dendritic morphology compared with 3‐month‐old mice. By dendrite reconstruction, we quantitatively assessed dendritic morphology of newborn immature neurons in the HDG of adult (*n* = 3, 46 neurons in total) and aged mice (*n* = 4, 91 neurons in total) (Fig. 1F,G). In 3‐month‐old mice, newborn neurons typically have 4.1 ± 0.4 dendritic branches with a total length of 297.0 ± 65.4 μm (Fig. 1H,I). In the 12‐month‐old cohort, newborn neuron dendritic number dramatically fell by 58% to 1.7 ± 0.2 per neuron (*P* = 0.004, Fig. 1H), while total dendritic length was significantly decreased to 70.6 ± 18.2 μm per neuron (*P* = 0.022, Fig. 1I), representing a 76% drop. Collectively, we observed dramatically decreased newborn immature neuron number and impaired dendritic morphology in the newborn neurons in the HDG of the aged mice, which recapitulates previous report in aged rats (Rao *et al*., 2006).

### The effect of DHF on newborn neuron survival in aged mice

So far, no effective drug exists for delaying or preventing age‐related cognitive decline. Developing approaches aimed at promoting neurogenesis and/or dendritic morphology is necessary. One potential target for therapeutic interventions is brain‐derived neurotrophic factor (BDNF), known to promote neuronal growth and survival and decrease in aging (Binder & Scharfman, 2004; Budni *et al*., 2015). However, the poor permeability through the blood–brain barrier and the potential immune reactivity largely limit direct application of BDNF. A small molecule, DHF, with high affinity for the BDNF receptor TrkB (Jang *et al*., 2010), is thus applied to activate BDNF signaling pathway in some circumstances (Choi *et al*., 2010; Liu *et al*., 2010; Andero *et al*., 2012; Zeng *et al*., 2012; Marongiu *et al*., 2013; Zhang *et al*., 2014). Previous studies in our group have proven the effects of DHF on BDNF receptor TrkB (Chen *et al*., 2015), and the beneficial roles of DHF on newborn immature neuron survival and dendritic morphology development in the HDG after traumatic brain injury (Zhao *et al*., 2015). Therefore, we assessed whether activation of BDNF signaling by applying DHF could also rescue the decline in neurogenesis and deficits of dendritic morphology caused by aging.

**Figure 2:**
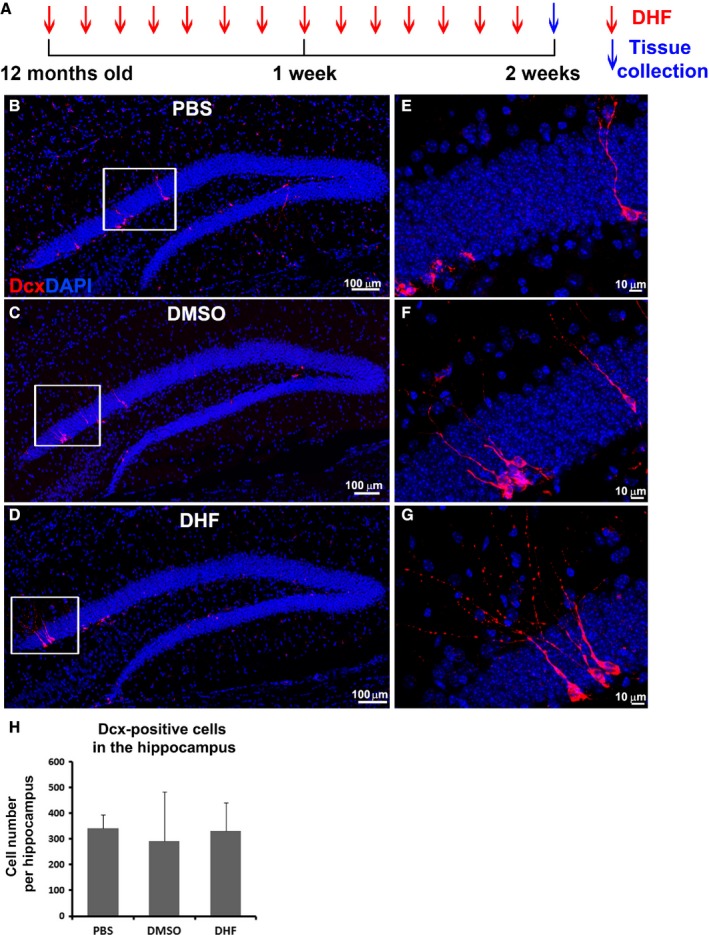
DHF treatment did not significantly increase newborn immature neuron number in the hippocampus in aged mice. (A) Schematic shows experimental strategy. (B–D) Immunostaining with doublecortin (Dcx, red) shows immature neurons in the hippocampal dentate gyrus (HDG) of 12‐month‐old mice that received phosphate‐buffered saline (PBS;* n* = 4, B), dimethyl sulfoxide (DMSO;* n* = 4, C), and 7, 8‐dihydroxyflavone (DHF;* n* = 4, D) treatments. 4′,6‐diamidino‐2‐phenylindole (DAPI, blue) staining shows the structure of HDG. (E‐G) Images of higher magnification from B‐D (indicated by white boxes) show individual Dcx‐positive immature neurons in the HDG of 12‐month‐old mice received PBS (E), DMSO (F), and DHF (G) treatments. (H) Quantification of Dcx‐positive immature neuron number in the HDG of 12‐month‐old mice received PBS, DMSO, and DHF treatments.

First, we sought to determine whether DHF promotes newborn neuron survival in the HDG of aged mice. Twelve‐month‐old mice received injections of DHF (5 mg kg^−1^ dissolved in dimethyl sulfoxide [DMSO], *n* = 4), 70% DMSO (*n* = 4), or phosphate‐buffered saline solution (PBS, *n* = 4) once daily for 2 weeks. All mice were sacrificed 24 h after the last injection, and brains were removed (Fig. 2A) for assessing the number of immature neurons and dendrite morphologies. Immunostaining of every one out of six brain sections was performed using the doublecortin (Dcx) antibody to detect newborn neurons in the hippocampus. In DMSO vehicle‐treated mice, we observed very few Dcx‐positive cells compared with PBS‐treated aged mice (Figs 1B and 2B,C). After DHF treatment, the number of Dcx‐positive cells did not show dramatic alteration (Fig. 2D). At higher magnification, we can count each single Dcx‐positive cell (Fig. 2E–G). The average number of Dcx‐positive cells per HDG in the DHF‐treated group was 330 ± 111, compared with 293 ± 191 in the DMSO vehicle group, and 341 ± 54 in the PBS control group (Fig. 2H). No statistic difference was detected among all three treated groups. Therefore, DHF treatment for 2 weeks did not increase newborn immature neuron survival in the HDG in aged mice. Our previous study showed that treatment with DHF for 2 weeks at the same dose increased the number of adult‐born immature neurons in the hippocampus of young adult animals (Zhao *et al*., 2015). Thus, longer treatment with DHF may be required to significantly promote immature neuron survival in the aging animal.

### DHF improved dendritic morphology in aged mice

**Figure 3:**
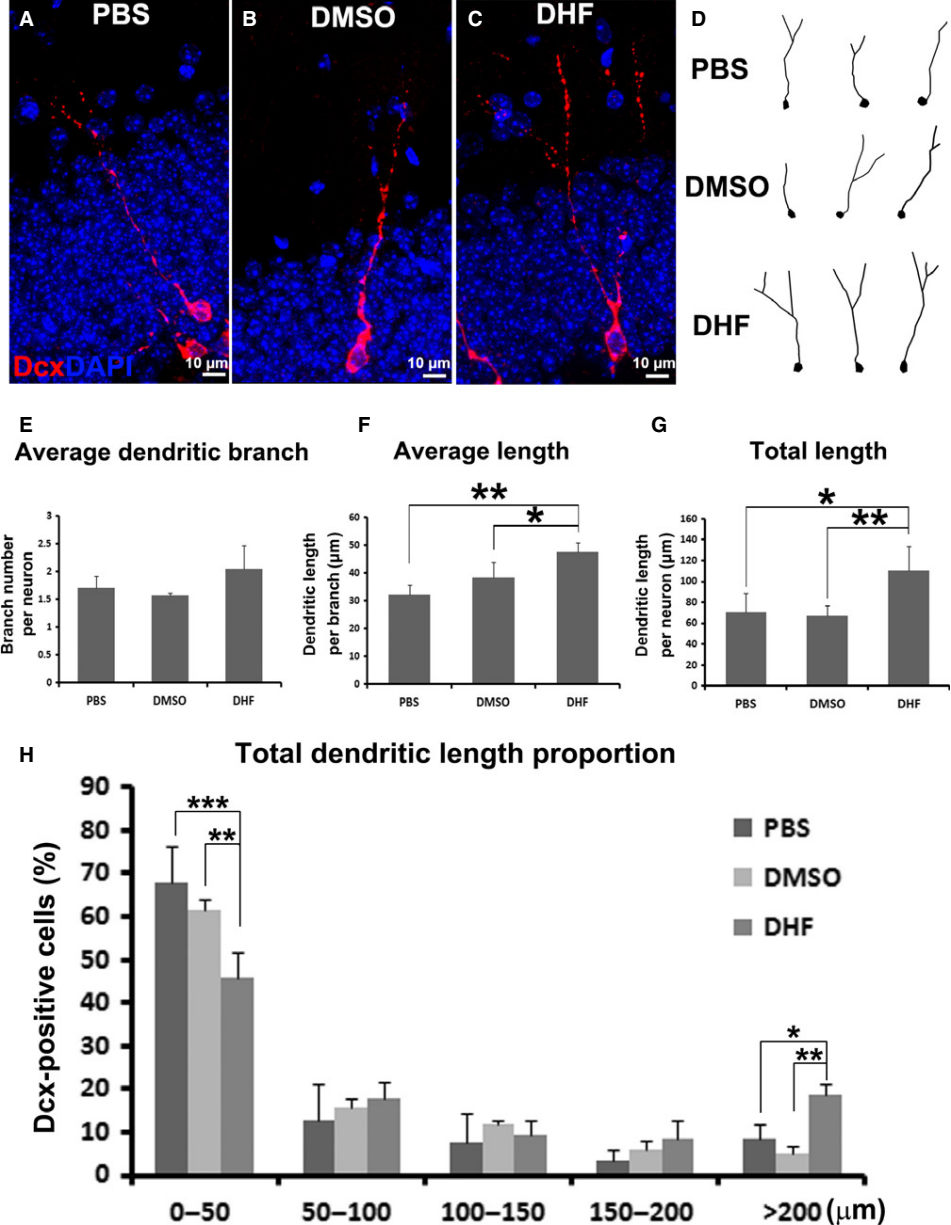
DHF treatment improved dendritic morphology development in newborn immature neurons in the hippocampus in aged mice. (A–C) Immunostaining with antibody against doublecortin (Dcx, red) shows structure of individual immature neurons in the hippocampal dentate gyrus (HDG) of 12‐month‐old mice received phosphate‐buffered saline (PBS;* n* = 4, B), dimethyl sulfoxide (DMSO;* n* = 4, C), and 7, 8‐dihydroxyflavone (DHF;* n* = 4, D) treatments. 4′,6‐diamidino‐2‐phenylindole (DAPI, blue) staining shows the structure of granule cell layer. (D) Reconstruction of dendrites from Dcx‐positive immature neurons in the HDG of 12‐month‐old mice received PBS, DMSO, and DHF treatments. (E–G) Quantifications of average dendritic branch number (E), average dendritic length (F), and total dendritic length (G) of Dcx‐positive immature neurons in the HDG of 12‐month‐old mice received PBS, DMSO, and DHF treatments. (H) Constituent ratio of Dcx‐positive immature neurons with total dendritic length in different categories in the HDG of 12‐month‐old mice received PBS, DMSO, and DHF treatments. (*P < 0.05, **P < 0.01, ***P < 0.001).

Although DHF did not increase newborn neuron survival in aged mice, microscopy revealed a change in their dendritic morphology (Figs 2E–G and 3A–C). Images at 40× magnification were taken of each hippocampal Dcx‐positive cell using the Zeiss microscopy system. The cell bodies and dendrites were then traced and reconstructed using Neurolucida software (Fig. 3D) and analyzed with NeuroExplorer (Fig. 3E–H). We found the average number of dendritic branch per neuron slightly increased from 1.7 ± 0.2 in the PBS group (*n* = 4, 91 neurons in total) and 1.6 ± 0.04 in the DMSO vehicle group (*n* = 4, 94 neurons in total) to 2.1 ± 0.4 in the DHF treatment group (*n* = 4, 109 neurons in total), but the difference was not significant (Fig. 3E). However, the average dendritic length per branch was dramatically increased from 32.1 ± 3.4 μm in PBS and 38.3 ± 5.5 μm in DMSO groups to 47.6 ± 3.4 μm after DHF treatment (Fig. 3F). As a result, total dendritic length per neuron was dramatically increased from 70.6 ± 18.2 μm in the PBS solution group and 67.0 ± 9.8 μm in the DMSO vehicle group to 110.3 ± 23.7 μm after DHF treatment (Fig. 3G). By further splitting newborn immature neurons into different categories according to their total dendritic length, we discovered in PBS and DMSO control groups, newborn immature neurons primarily develop very short dendrites within 50 μm to their soma in total (67.8 ± 8.8% in PBS mice, 61.4 ± 2.4% in DMSO mice, Fig. 3H). The percentage of newborn neurons with short total dendritic length within 50 μm was dramatically reduced to 45.9 ± 5.7% in the DHF group (Fig. 3H). Consequently, more newborn neurons in the DHF‐treated mice developed relatively long dendrites > 200 μm to their soma, accounting for 18.7 ± 2.6% in all the measured newborn neurons compared with only 8.4 ± 3.3% in the PBS group and 5.1 ± 2.0% in the DMSO group (Fig. 3H). Collectively, although DHF did not promote newborn immature neurons to grow more dendritic branches, it instead improved dendritic development by enhancing dendritic elongation shown as increased average and total dendritic length in aged mice, as well as shifting the percentage of newborn immature neurons toward the category with longer total dendrites at a population level.

## Discussion

As the aged population grows rapidly, age‐dependent cognitive decline will become an increasing problem for our society (Ortman *et al*., 2014). However, the molecular mechanism of this decline is largely unknown, hindering development of a therapy to delay or prevent it. Neuroplasticity, represented by hippocampal neurogenesis and synaptic plasticity, which are important events for learning and memory capacity, was reported to be compromised as age advances in rodents and humans (Burke & Barnes, 2006; Rao *et al*., 2006). In the present study, we used 12‐month‐old mice and observed some of the characteristics previously seen in aging subjects, most notably decreased newborn immature neuron number and impaired dendritic morphology in those newborn neurons, compared with 3‐month‐old young adult cohort (Fig. 1). These observations represent deficits in neurogenesis and synaptic plasticity in the aging group. Approaches aimed at neurogenesis and/or synaptic plasticity enhancement might be potential therapeutic targets.

So far, no effective treatment is available to enhance neurogenesis or synaptic plasticity for aged subjects. Exercise has been shown to be beneficial to overall general health (Kempermann *et al*., 1997, 1998; Kempermann & Gage, 1999; van Praag *et al*., 1999a,b; Cotman & Berchtold, 2002). The running mice perform much better than sedentary mice in learning tasks, for example, spatial learning. The more complex the tasks are, the better the exercising mice perform (van Praag *et al*., 1999a, 2000). It has been shown that physical exercise moderately enhances neurogenesis in the adult hippocampus (van Praag *et al*., 1999b, 2005; Kempermann *et al*., 2000) and improves functional performance following TBI (Wagner *et al*., 2002; Kline *et al*., 2007; Hoffman *et al*., 2008), whereas brain‐derived neurotrophic factor (BDNF) has been found to enhance the survival and dendritic growth of newborn neurons in the hippocampus (Tolwani *et al*., 2002; Wang *et al*., 2015) and may be potentially beneficial for neurogenesis and/or synaptic plasticity in aged subjects as well. A small‐molecule BDNF receptor agonist, DHF (Jang *et al*., 2010), surpasses BDNF due to its permeability through the blood–brain barrier and low immune reactivity when alternatively applied to activate BDNF signaling. In previous studies, we have proven the beneficial roles of DHF pre‐ or post‐treatment in the case of traumatic brain injury by activating the BDNF receptor TrkB, promoting newborn neuron survival, and enhancing newborn neuron dendritic development (Chen *et al*., 2015; Zhao *et al*., 2015). In the present study, we proposed that the beneficial roles of DHF would also improve neurogenesis and/or synaptic plasticity in the case of aging. We applied DHF for 2 weeks, and surprisingly, it did not significantly increase newborn neuron survival in aged mice (Fig. 2). However, we did discover extensive dendritic morphological changes between the control and treatment groups. Our data suggested that by daily injection of DHF for 2 weeks, although dendritic branch number of newborn neurons was not significantly altered, the average dendritic length of newborn neurons was dramatically increased by 48% compared with the PBS group and by 24% compared with the DMSO vehicle group (Fig. 3F). Similarly, total dendritic length of newborn neurons was dramatically increased by 53% compared with the PBS group and by 64% compared with the DMSO vehicle group (Fig. 3G). Additionally, we observed a shift of the constituent ratio of newborn neurons toward the category with longer dendrites, shown as a 2.2‐fold increase in the proportion of neurons with total dendrites longer than 200 μm (Fig. 3H).

Similarly, Zhang *et al*. (2014) discovered that exposure to 500 nm DHF for 3 days increased total dendrite length and number in cultured neurons, and Zeng *et al*. (2012) found that spine density and number were markedly increased in 22‐month‐old rats treated with DHF daily for 34 days. In the 30‐month‐old rats that received an identical treatment, DHF restored spine density but not dendrite number (Zeng *et al*., 2012). Although differences exist among studies, possibly resulting from varying treatment protocols and ages of animals, overall improvements in dendritic morphology were observed from various perspectives. Dendrites form synapses with axons from surrounding neurons and transmit information to the neuron's cell body. A decline in the length and number of dendrites available to receive and transmit information impairs neuronal signaling in aged subjects, while DHF improved dendritic length, which serves as a foundation for potential gain of synapses and restoration of signaling transmission.

BDNF has been shown to control the shape and number of dendritic spines, thus influencing the development of synaptic circuits particularly in the hippocampus (Ji *et al*., 2005; Cohen‐Cory *et al*., 2010). Our previous study directly demonstrated that BDNF knockout impaired synapses formation in postnatal granule cells in hippocampal dentate gyrus (Gao *et al*., 2009). Here, we showed that the total length of dendrites of each newborn neuron dramatically reduced with aging.

The total length of dendrites in each neuron reduced to 23.7% from a mouse aged 3 to 12 months. DHF treatment in the present study significantly increased both the average and the total length of dendrites in each newborn neuron in the aged mouse. However, compared with the 3‐month‐old young adult cohort, the dendrites in aging mice after DHF treatment were still much shorter, representing only 37% of the adult level at the age of 3 months old. Additionally, we did not evaluate dendritic spine and synapse formation on the elongated dendrites. Although longer dendrites provide larger surface area and thus may allow neurons to form more synapses with upstream neurons, amplification of dendritic length in hippocampal granule neurons does not necessarily guarantee synaptogenesis and consequent improvement in cognition in aged mice. Further investigations are needed to explore the effect of DHF on mature neuron functions and behavioral performances in aged animals.

**Figure 4:**
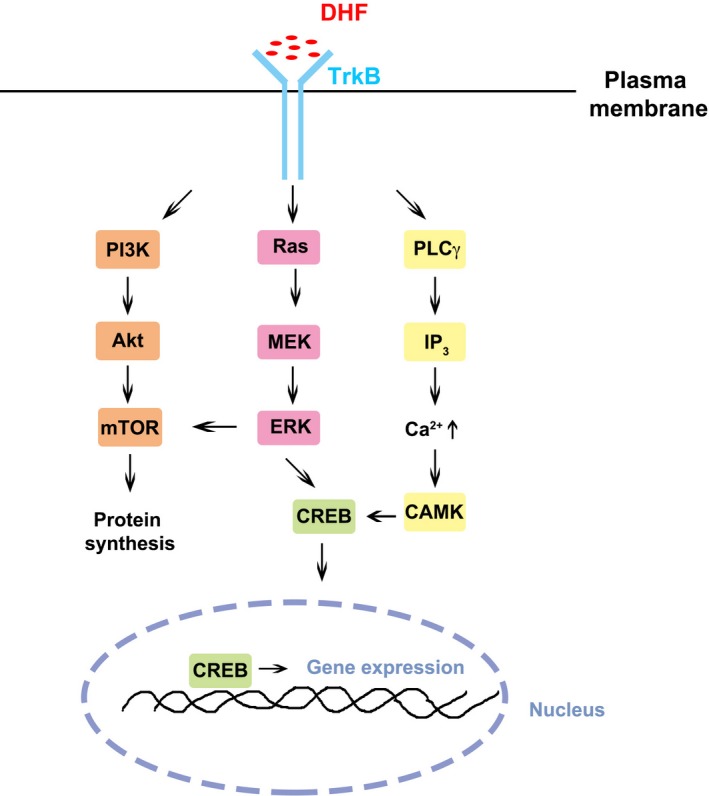
Potential mechanisms involved in DHF‐improved dendritic morphology development. Intracellular signaling pathways that have been reported to be involved in BDNF‐TrkB system activation. Downstream effects may consist of mTOR activation and following global protein synthesis, as well as CREB phosphorylation by ERK‐ or calcium‐activated CAMK and subsequent CREB nuclear translocation‐activated gene expression. PI3K: Phosphoinositide 3‐kinase; Akt: Protein kinase B; mTOR: Mammalian target of rapamycin; CREB: cAMP response element‐binding protein; PLCγ: Phospholipase C γ; IP3: Inositol triphosphate; CAMK: Calcium‐/calmodulin‐dependent protein kinase.

BDNF expression level has been reported to be decreased in the hippocampus in aged rats (Calabrese *et al*., 2013) and thus was considered a potential reason for age‐dependent neurogenesis and dendritic impairments. Aimed at this issue, we treated 12‐month‐old mice with DHF to activate BDNF signaling and observed improved dendritic morphology in the aged mice, while the molecular mechanism is still largely unknown. Numerous studies have also highlighted the ability of DHF to influence dendrite synapse density through TrkB upregulation (Luine & Frankfurt, 2013; Castello *et al*., 2014); however, little research has been performed on the downstream effects of DHF on signaling pathway regulation and/or gene expression level. Several ubiquitous intracellular signaling pathways have been reported to be involved in BDNF‐TrkB system activation, including PI3K/Akt, Ras/MEK/ERK, and PLCγ/IP3 signaling (Chao, 2003; Duman & Voleti, 2012; Russo & Nestler, 2013). Downstream effects may consist of mTOR activation and following global protein synthesis, as well as CREB phosphorylation by ERK‐ or calcium‐activated CAMK and subsequent CREB nuclear translocation‐activated gene expression (Fig. 4). As TrkB agonist imitating BDNF actions, DHF potentially benefits dendritic morphology development by modulating these signaling pathways in aged subjects and thus may potentially improve synaptic transmission and cognitive outcomes in aged subjects. Further investigations are needed to elucidate the mechanisms and to facilitate therapeutic applications of DHF to improve cognitive functions in the aged population.

## Experimental procedures

### Animal care

Male C57 BL/6 mice (Jackson Laboratories) were group‐housed and kept in a 12‐h/12‐h light/dark cycle with free to access to food and water at all times. The animals were used in experiments at ages of 3 and 12 months. All procedures were performed under protocols approved by Indiana University's Animal Care and Use Committee.

### DHF treatment

Mice at the age of 12 months received either DHF (5 mg kg^−1^ as applied in previous study (Zhao *et al*., 2015), dissolved in dimethyl sulfoxide [DMSO], *n* = 4), 70% DMSO (*n* = 4), or phosphate‐buffered solution (PBS, 50 mg kg^−1^, *n* = 4, intraperitoneal [i.p.]) injections once a day for 2 weeks. Twenty‐four hours after the last injection, the mice were sacrificed for quantifying newborn neurons and assessing newborn neuron dendrite morphology (Fig. 2A).

### Tissue processing

Animals were deeply anesthetized with 2.5% avertin and then were perfused transcardially with cold 0.9% saline, followed by a cold fixative containing 4% paraformaldehyde (PFA) in PBS. The brains were removed, postfixed in PFA overnight, and cryoprotected with 30% sucrose for 48 h. Serial coronal sections (30 μm thick) were cut using a cryostat (Leica, CM1900, Wetzlar, Germany) and stored at −20 °C. The sections were then processed for immunohistochemical analysis.

### Immunohistochemistry

Series of every sixth section (180 μm apart) through each hippocampus were processed with standard floating sections immunostaining. Each section was washed with PBS three times for 30 min. The sections were blocked in PBS containing 5% normal goat serum, 0.1% bovine serum albumin (BSA), and 0.1% Triton x‐100. Antidoublecortin (Dcx) primary antibody (1:1000, guinea pig; EMD Millipore, Darmstadt, Germany) was applied overnight at 4 °C. Then, goat anti‐guinea pig secondary antibody from Jackson Immunoresearch was applied at 1:1000 dilution.

### Cell counting

Immunohistochemistry was performed simultaneously on sections to detect the Dcx‐positive cells. Series of every sixth section (30 μm thick, 180 μm apart) through each hippocampus were processed. The cell number was determined through a blinded quantitative histological analysis under a profile count protocol as described previously (Gao & Chen, 2013). Briefly, every single Dcx‐positive cell was counted throughout the granular cell layer (GCL) in the 30‐μm section in multiplanes.

### Microscopy

The sections were analyzed using an inverted microscopy system (Zeiss Axiovert 200M, Oberkochen, Germany) combined with ApoTome and interfaced with a digital camera (Zeiss AxioCam MRc5) controlled by a computer. Images were captured using ApoTome in conjunction with axiovision v4.8 software (Zeiss AxioVision, v4.8) and then assembled and labeled in photoshop 7.0 (Adobe Systems, San Jose, CA).

### Dendrite tracing and analysis

40× images of each individual Dcx‐positive cell, which has dendritic structure, were captured and used to trace neuron cell bodies and dendrites using the neurolucida software (MBF Bioscience, Williston, VT, USA). The neuron tracings were then analyzed on neuroexplorer software (Next Technologies, Madison, AL, USA). In 3‐month‐old adult mice, 46 neurons in total (*n* = 3) were analyzed. In 12‐month‐old mice, 91 neurons in total (*n* = 4), 94 neurons in total (*n* = 4), and 109 neurons in total (*n* = 4) were assessed in PBS, DMSO, and DHF groups, respectively.

### Statistical analysis

The collected data were expressed as average ± standard deviation. Data of comparison on newborn immature neuron number, average dendritic number, and total dendritic length in 3‐month‐old and 12‐month‐old mice were analyzed using Student's *t*‐test (Fig. 1E,H,I). Results on newborn immature neuron number, average dendritic number, average dendritic length, and total dendritic length after PBS, DMSO, and DHF treatments in 12‐month‐old mice (Figs 2H and 3E–G) were analyzed by one‐way analysis of variance (anova) followed by Fisher's least significant difference (LSD) as *post hoc* test. Data of constituent ratio of newborn immature neuron total dendritic length in different categories in 12‐month‐old mice that received PBS, DMSO, and DHF treatments (Fig. 3H) were analyzed via two‐way anova followed by one‐way anova with LSD as *post hoc* test. Statistical analysis were performed using spss software (IBM Cooperation, Armonk, NY) with significance set at *P* < 0.05.

## Funding

This work was supported by grants from NIH 1R21NS072631‐01A (JC) and the Indiana Spinal Cord & Brain Injury Research Grants (JC).

## Author contributions

X.W., J.L.R., and X.G. performed experiments, analyzed data, and wrote manuscript; J.C. designed experiment and wrote manuscript.

## Conflict of interest

None declared.

## Supporting information


**Fig. S1** Large majority of Dcx‐positive cells in the aged hippocampus are postmitotic immature neurons.
