# Supplementary figures and images for: Aging impairs dendrite morphogenesis of newborn neurons and is rescued by 7, 8‐dihydroxyflavone
Source: Aging Cell. 2017 Mar 3;16(2):304–11. doi: 10.1111/acel.12553 (PMC5334527; doi:10.1111/acel.12553)

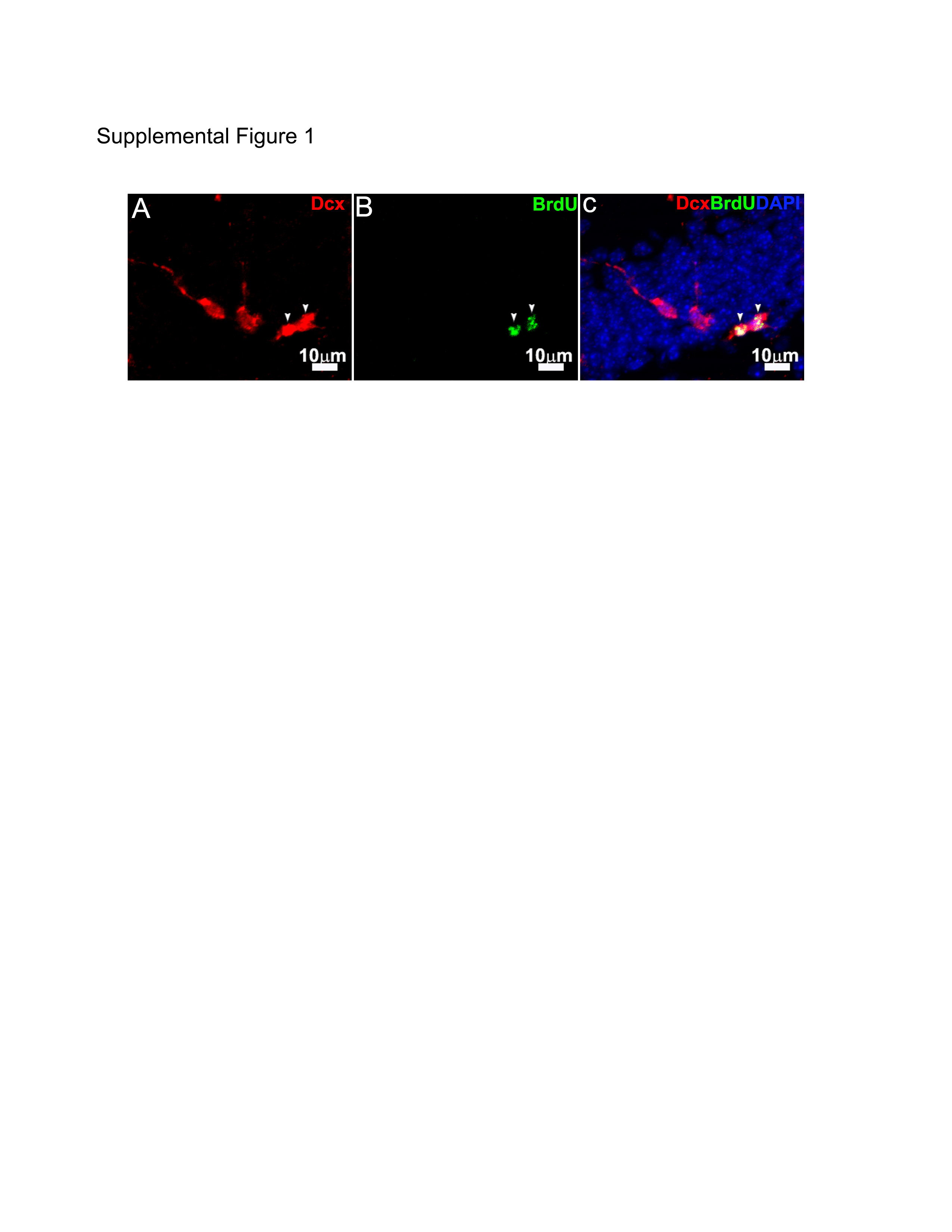

Supplement: Supplementary file 1 — Fig. S1 Large majority of Dcx‐positive cells in the aged hippocampus are postmitotic immature neurons. [file ACEL-16-304-s001.jpg]
